# Transactions

**Published:** 1880-06

**Authors:** 


### TRANSACTIONS.

The volume of transactions of the last annual meeting of the Ohio State Dental Society, was issued recently by Ransom & Randolph, of Toledo, Ohio. It contains ninety-two pages, and is well filled with interesting matter in discussions and essays, and is well worth perusal by every dentist.

It can be obtained of the publishers, Ransom & Randolph, or of the editor of the *Register.* Send postage.

